# Supplementary material for: From moral distress to resilient ethical climate among general practitioners: Fostering awareness. A qualitative pilot study
Source: PLoS One. 2024 Aug 30;19(8):e0306026. doi: 10.1371/journal.pone.0306026 (PMC11364290; doi:10.1371/journal.pone.0306026)
Supplement: S1 File — (DOCX) [file pone.0306026.s001.docx]

**Supporting Information File 1. Interview Guide.**

## **Methodology**

We prepared these interviews using the "Manual of Qualitative Research Methods" (1) as a guide and processed them using the "Qualitative Analysis Guide of Leuven" (QUAGOL) (2).

We based the questions of the interview schedule on the available literature as briefly described in the introduction. The guiding questions were similar to the research questions:

- How does moral distress manifest itself in Flemish general practice?

- What is the role of the ethical climate in the experience of moral distress among Flemish GPs?

- How can ethical climate and moral distress result in moral resilience in Flemish GP practice?

The interview started from a concrete experience of moral stress by the interviewee to explore further the ethical climate and moral resilience.

This interview schedule was tested in a practice interview with an independent doctor, after which it was further refined, among other things in terms of interview style. The questionnaire used at the start of the in-depth interviews (see below) was used flexibly throughout the course of the interviews and adapted on the basis of respondent input in an iterative process.

Respondents for the interviews were recruited after completing a survey, where they could leave their contact details if they were interested in participating in the in-depth interviews. As the number of participants in the in-depth interviews was initially limited and not representative of the entire study population, we further opted to invite individual doctors personally to take part by e-mail or telephone.

Informed consent was obtained from all participants before starting these interviews.

One of the two investigators (RC or AS) interviewed the chosen physician each time. During the Covid19 pandemic we were forced to conduct these interviews mainly via video interview (Zoom). The interviews took place between 24 April 2021 and 12 October 2021, lasted for an average of 1 hour, were audio-recorded integrally and anonymously, and were then transcribed ad verbatim.

(1) Mortelmans D. Handboek kwalitatieve onderzoeksmethoden. Blijde Inkomstraat 22, 3000 Leuven (België): UItgeverij Acco; 2007. 534 p.

(2) Dierckx de Casterlé B, Gastmans C, Bryon E, Denier Y. QUAGOL: a guide for qualitative data analysis. Int J Nurs Stud. 2012;49(3):360-71.

## **Invitation**

After analysing the questionnaire you filled in, we will go deeper into the concrete and personal experience of moral stress, ethical climate and their interrelation in general practice. We do this by conducting in-depth interviews. Participation in these in-depth interviews takes about 1 hour, at a time that suits you. If you are interested in this, you can leave your contact details using the questions below.

We will only use these details to make further arrangements with you for our in-depth interview and to keep you further informed.

Name:

________________________________________________________________

Email address:

________________________________________________________________

Optional: phone number:

________________________________________________________________

## **Interview scheme**

***Objective/Core questions***

- How does moral stress manifest itself in the practice of Flemish general practitioners?
  - Which concrete situations cause moral distress among Flemish GPs?
  - What is the importance of moral stress for Flemish GPs?
  - How do Flemish GPs react when they experience moral distress?
- What is the role of the ethical climate in the experience of moral stress among Flemish GPs?
  - How do Flemish GPs approach the ethical climate within their practice?
  - What is the importance of ethical climate in Flemish general practice?
- How can ethical climate and moral stress contribute to moral resilience in Flemish general practice?

Opening

- Welcome, interviewer introduces himself
- Clarification of the duration, purpose and importance of this interview
- Informed consent/confidentiality

Interview

Can you think of a situation (or several situations) in which you experienced a sense of bitterness because you could not act according to your moral values and according to what you thought was the right thing to do? This is because you were prevented from doing so by internal and external factors.

- Distinguish from ethical dilemma where it is impossible to make an ethically "correct" choice due to a conflict of values

*Facts*

- Describe the story.
- Which parties were involved?
- Were there any alternatives to the created situation?
- Were there any influencing factors, barriers or impediments?
  - With yourself?
  - Within the organisation where you work?
  - In a wider context?
- Did the story end well or badly? What made it end this way? What could have been a positive/negative outcome?

*Feelings and values*

- How did you feel at that time?
- What values were at play at that time (which party has which value)?
- Why exactly did you feel (not) good about the situation? Did you feel stress?

*Elaboration of the situation*

Talking about it

- Mechanisms of cooperation in the event of ethical difficulties?
  - How do you feel about the presence or absence of these?
- Discussion on the workfloor? Do you feel you can do something about it? (Trust, power)
- Reaction of colleagues (openness, flexibility of role, inclusion; willingness to reach a consensus)
- How did it make you feel?
- How could this have been done better?
- How are differences dealt with?

Coping

- How did you deal with the situation (other than talking about it)?
  - In the short term? In the long term?
  - Could you let off steam when you felt bad about the situation?
    - Talk about it? Colleagues? At Home? Friends?
    - Hobbies: Sports? Music?
    - Others? Self-esteem? Self-compassion? Humour (personal resources)?
- How did colleagues deal with the situation?
- What was the effect of this event on professional identity and functioning as a doctor in you and in colleagues?
  - Moral resilience?
  - "moral residue?" and crescendo effect?
  - Job abandonment?

*Wider framework and ethical environment*

- - When problems like the case described arise, who takes the lead in this? (Ethical leadership).
    - How is this done?
    - Is there someone who takes the lead in this process? Adjustment, training, point of contact
    - Were you the driving force to work on this? Why or why not?
  - Does your partnership have a vision or mission of practice to fall back on in such situations? (Organisational culture; shared narrative)
    - Explicit or implicit? Carried vision or "scrap of paper"? (Collective moral motivation, moral character)
    - Is it regularly reviewed?
    - Is it based on values?
    - Are there also people in the organisation who are not believing in this vision?
  - How is the atmosphere between colleagues in practice? (openness, trust etc)
    - A lot of dedication? Close-knit group? A lot of absenteeism? Humanity? Empathy?
  - What is the influence of just described phenomena (practice vision, leadership, atmosphere) on your moral stress and actions in practice? (ethical climate as mediator)
- **Moral assessment**
  - If ethical climate is present: what happens if consensus is not reached immediately in a given situation?
  - o Trust, openness, curiosity, inclusiveness, will to reach consensus?
- **Moral motivation and moral character**
  - Is there the possibility to make time for further training/depth work in case of moral stress or ethical problems? (Inspired staff).
  - Do you and/or your colleagues succeed in translating what is discussed into action? Why or why not? (Moral character)
  - Which mechanisms help or block to put the made moral decision into practice? (moral character)

*Moral resilience/moral success*

- What opportunities do you see within your organisation and from the experience described to turn moral stress into something positive?
- What factors led you to believe that you could find a solution to moral stress? ("power", "trust")
- What is a positive outcome?

*Ethical ambitions*

- Try to define an ethical climate in which you would prefer to work.
- (How) does this differ from the current situation?

Conclusion

- *Brief summary of what was discussed during the interview (by the interviewer).*
- *Bringing up topics that were not discussed but that the doctor expected.*
- *Which of the elements discussed did you find most important?*
- *Do you still have the same opinion about this topic as before our interview?*
- *Have we overlooked anything regarding this interview? Is there anything else you would like to add?*
- *Thank you*

1. **Reference list**

**The following list of references inspired this interview guide. References marked in bold are considered to have contributed most to our interview guide:**

**Koskenvuori J, Numminen O, Suhonen R. Ethical climate in nursing environment: A scoping review. Nurs Ethics. 2019;26(2):327-45.**

Hamric AB, Blackhall LJ. Nurse-physician perspectives on the care of dying patients in intensive care units: collaboration, moral distress, and ethical climate. Crit Care Med. 2007;35(2):422-9.

Whitehead PB, Herbertson RK, Hamric AB, Epstein EG, Fisher JM. Moral distress among healthcare professionals: report of an institution-wide survey. J Nurs Scholarsh. 2015;47(2):117-Morley G, Ives J, Bradbury-Jones C, Irvine F. What is 'moral distress'? A narrative synthesis of the literature. Nurs Ethics. 2019;26(3):646-62.

**Denier Y, Gastmans C, Cloet M. Zorgnet-Icuro. Ethisch Advies 21 - Morele Stress in de zorg. Brussel2019.**

Vanlaere L, Gastmans C. Ethics in Nursing Education: Learning To Reflect On Care Practices. Nursing Ethics. 2007;14(6):758-66.

Jameton A. Dilemmas of moral distress: moral responsibility and nursing practice. AWHONNS Clin Issues Perinat Womens Health Nurs. 1993;4(4):542-51.

**Kherbache A, Mertens E, Denier Y. Moral distress in medicine: An ethical analysis. J Health Psychol. 2021.**

Bollen M, Pieters M-H, Denier Y. Moral distress among care providers in the residential care of elderly: a qualitative case-study. Leuven: KU Leuven; 2018.

Pauly B, Varcoe C, Storch J, Newton L. Registered nurses' perceptions of moral distress and ethical climate. Nurs Ethics. 2009;16(5):561-73.

**Epstein EG, Whitehead PB, Prompahakul C, Thacker LR, Hamric AB. Enhancing Understanding of Moral Distress: The Measure of Moral Distress for Health Care Professionals. AJOB Empir Bioeth. 2019;10(2):113-24.**

Webster G, Baylis F, Rubin S, Zoloth L. Margin of error: the ethics of mistakes in the practice of medicine. Haggerstown: University Publishing Group. 2000:217-30.

Lamiani G, Borghi L, Argentero P. When healthcare professionals cannot do the right thing: A systematic review of moral distress and its correlates. J Health Psychol. 2017;22(1):51-67.

**Epstein EG, Hamric AB. Moral distress, moral residue, and the crescendo effect. J Clin Ethics. 2009;20(4):330-42.**

Corley MC, Elswick RK, Gorman M, Clor T. Development and evaluation of a moral distress scale. J Adv Nurs. 2001;33(2):250-6.

Hamric AB. Moral distress and nurse-physician relationships. Virtual Mentor. 2010;12(1):6-11.

**Van Daele L, Van De Vijver L, Jadoul C, De La Meilleure G. Eerste hulp bij ethische stress. Tijdschrift Voor Geneeskunde. 2019;75(6):400-8**.

Rushton CH. Moral Resilience: A Capacity for Navigating Moral Distress in Critical Care. AACN Adv Crit Care. 2016;27(1):111-9.

**Rushton CH, Caldwell M, Kurtz M. Moral Distress: A Catalyst in Building Moral Resilience. Am J Nurs. 2016;116(7):40-9.**

Sundin-Huard D, Fahy K. Moral distress, advocacy and burnout: theorizing the relationships. Int J Nurs Pract. 1999;5(1):8-13.

Shoorideh FA, Ashktorab T, Yaghmaei F, Alavi Majd H. Relationship between ICU nurses' moral distress with burnout and anticipated turnover. Nurs Ethics. 2015;22(1):64-76.

Dzeng E, Curtis JR. Understanding ethical climate, moral distress, and burnout: a novel tool and a conceptual framework. BMJ Qual Saf. 272018. p. 766-70.

Dzeng E, Wachter RM. Ethics in Conflict: Moral Distress as a Root Cause of Burnout. J Gen Intern Med. 352020. p. 409-11.

Delfrate F, Ferrara P, Spotti D, Terzoni S, Lamiani G, Canciani E, et al. Moral Distress (MD) and burnout in mental health nurses: a multicenter survey. Med Lav. 2018;109(2):97-109.

Dalmolin Gde L, Lunardi VL, Lunardi GL, Barlem EL, Silveira RS. Moral distress and burnout syndrome: are there relationships between these phenomena in nursing workers? Rev Lat Am Enfermagem. 2014;22(1):35-42.

Shanafelt TD, Dyrbye LN, West CP. Addressing Physician Burnout: The Way Forward. Jama. 2017;317(9):901-2.

Shanafelt TD, Noseworthy JH. Executive Leadership and Physician Well-being: Nine Organizational Strategies to Promote Engagement and Reduce Burnout. Mayo Clin Proc. 2017;92(1):129-46.

Shanafelt T, Swensen S. Leadership and Physician Burnout: Using the Annual Review to Reduce Burnout and Promote Engagement. Am J Med Qual. 2017;32(5):563-5.

Panagioti M, Geraghty K, Johnson J, Zhou A, Panagopoulou E, Chew-Graham C, et al. Association Between Physician Burnout and Patient Safety, Professionalism, and Patient Satisfaction: A Systematic Review and Meta-analysis. JAMA Intern Med. 2018;178(10):1317-30.

**Varcoe C, Pauly B, Webster G, Storch J. Moral distress: tensions as springboards for action. HEC Forum. 2012;24(1):51-62**.

Rushton CH, Carse A. Towards a New Narrative of Moral Distress: Realizing the Potential of Resilience. J Clin Ethics. 2016;27(3):214-8.

**Young PD, Rushton CH. A concept analysis of moral resilience. Nurs Outlook. 2017;65(5):579-87.**

Musto LC, Rodney PA. Moving from conceptual ambiguity to knowledgeable action: using a critical realist approach to studying moral distress. Nurs Philos. 2016;17(2):75-87.

Rest JR. The major components of morality. New York: John Wiley; 1984.

Stutzer K, Bylone M. Building Moral Resilience. Crit Care Nurse. 2018;38(1):77-89.

Meyers C. Institutional culture and individual behavior: creating an ethical environment. Sci Eng Ethics. 2004;10(2):269-76.

Victor B, Cullen J. The Organizational Bases of Ethical Work Climates. Administrative Science Quarterly. 1988;33:101-25.

**Olson LL. Hospital nurses' perceptions of the ethical climate of their work setting. Image J Nurs Sch. 1998;30(4):345-9.**

**Grönlund CF, Söderberg A, Dahlqvist V, Andersson L, Isaksson U. Development, validity and reliability testing the Swedish Ethical Climate Questionnaire. Nurs Ethics. 2019;26(7-8):2482-93.**

**Arnaud A. Conceptualizing and Measuring Ethical Work Climate: Development and Validation of the Ethical Climate Index. Business & Society. 2010;49(2):345-58.**

Ulrich C, Grady C. Moral Distress in the Health Professions: Springer International Publishing AG; 2018.

Smith KV. Ethical decision-making in nursing: implications for continuing education. J Contin Educ Nurs. 1996;27(1):42-5.

**Newman A, Round H, Bhattacharya S, Roy A. Ethical Climates in Organizations: A Review and Research Agenda. Business Ethics Quarterly. 2017;27(4):475-512**.

Goldman A, Tabak N. Perception of ethical climate and its relationship to nurses' demographic characteristics and job satisfaction. Nurs Ethics. 2010;17(2):233-46.

Abou Hashish EA. Relationship between ethical work climate and nurses' perception of organizational support, commitment, job satisfaction and turnover intent. Nurs Ethics. 2017;24(2):151-66.

Asgari S, Shafipour V, Taraghi Z, Yazdani-Charati J. Relationship between moral distress and ethical climate with job satisfaction in nurses. Nurs Ethics. 2019;26(2):346-56.

Huang CC, You CS, Tsai MT. A multidimensional analysis of ethical climate, job satisfaction, organizational commitment, and organizational citizenship behaviors. Nurs Ethics. 2012;19(4):513-29.

Dinc MS, Huric A. The impact of ethical climate types on nurses’ behaviors in Bosnia and Herzegovina. Nursing Ethics. 2017;24(8):922-35.

Van den Bulcke B, Metaxa V, Reyners AK, Rusinova K, Jensen HI, Malmgren J, et al. Ethical climate and intention to leave among critical care clinicians: an observational study in 68 intensive care units across Europe and the United States. Intensive Care Med. 2020;46(1):46-56.

Benoit DD, Jensen HI, Malmgren J, Metaxa V, Reyners AK, Darmon M, et al. Outcome in patients perceived as receiving excessive care across different ethical climates: a prospective study in 68 intensive care units in Europe and the USA. Intensive Care Med. 2018;44(7):1039-49.

Saygili M, Özer Ö, Karakaya P. Paternalistic Leadership, Ethical Climate and Performance in Health Staff. Hosp Top. 2020;98(1):26-35.

Suhonen R, Stolt M, Gustafsson ML, Katajisto J, Charalambous A. The associations among the ethical climate, the professional practice environment and individualized care in care settings for older people. J Adv Nurs. 2014;70(6):1356-68.

Ulrich C, Grady C. Moral Distress in the Health Professions: Springer International Publishing AG; 2018. 171 p.

Corley MC, Minick P, Elswick RK, Jacobs M. Nurse moral distress and ethical work environment. Nurs Ethics. 2005;12(4):381-90.

**Hamric AB, Borchers CT, Epstein EG. Development and Testing of an Instrument to Measure Moral Distress in Healthcare Professionals. AJOB Primary Research. 2012;3(2):1-9.**

Pendry PS. Moral distress: recognizing it to retain nurses. Nurs Econ. 2007;25(4):217-21.

Lützén K, Ewalds-Kvist B. Moral distress and its interconnection with moral sensitivity and moral resilience: viewed from the philosophy of Viktor E. Frankl. J Bioeth Inq. 2013;10(3):317-24.

Aydan S, Kaya S. Ethical climate as a moderator between organizational trust and whistle-blowing among nurses and secretaries. Pak J Med Sci. 2018;34(2):429-34.

**Mortelmans D. Handboek kwalitatieve onderzoeksmethoden. Blijde Inkomstraat 22, 3000 Leuven (België): UItgeverij Acco; 2007. 534 p.**
