# Supplementary material for: From moral distress to resilient ethical climate among general practitioners: Fostering awareness. A qualitative pilot study
Source: PLoS One. 2024 Aug 30;19(8):e0306026. doi: 10.1371/journal.pone.0306026 (PMC11364290; doi:10.1371/journal.pone.0306026)
Supplement: S2 File — (DOCX) [file pone.0306026.s002.docx]

**Supporting Information File 2. Conceptual interview schemes.**

# Interview 1

- Moral distress
  - Situation
    - Medical error
  - Determinants
    - Internal: Uncertainty about own ability
    - External:
      - Family
      - Good doctor-patient relationship
  - Importance
    - Strong emotions
    - Resilience
      - Dare to show vulnerability
      - Increased alertness
  - Coping:
    - Sports
    - Talking to patient and patient family
    - Talking to own relatives
    - Work harder
- Ethical climate
  - Respect
  - Humour
  - Cheerfulness
  - Following the heart
  - Scientifically correct medicine
  - Discussion in group
  - Value-driven cooperation

# Interview 2

- Moral distress
  - Situations
    - End of life care
    - Lack of communication
    - Fear of sharing own opinions
    - Perform treatments you do not support yourself
  - Determinants
    - Internal
      - Uncertain personality
    - External
      - Hierarchy: GPT vs GP
      - Covid
      - Shortage of straff
  - Effects:
    - Fear
    - Sorrow
    - Doubt
  - Coping
    - Sports
    - Avoidance
    - Talking to colleagues
- Ethical climate
  - Patient-centred care
  - Shared-decision making
  - Contact person for problems
  - Confidential advisor
  - Safety and security
  - Value-driven
  - Group discussion
  - Authenticity

# Interview 3

- Moral distress
  - Situations
    - Little communication
    - Ethical solitude
    - Narrative ethics
  - Determinants
    - Internal
      - Fear of own opinion
    - External
      - Shortage of resources/staff
      - Hierarchy
      - Intimidation
      - Lack of group discussion
  - Effects
    - Anger
    - Indignation
    - Strong concern about care of patients
    - Ethical stillness
  - Coping
    - Walking
    - Talking to relatives
- Ethical climate
  - Physician autonomy
  - Respect / dignity
  - Solidarity
  - Neutral and trained person of contact
  - Organisation from the GP association
  - Ventilating

# Interview 4

- Moral distress
  - Situations
    - Too much administration
    - Lack of continuity of care
    - Authority hierarchy
    - Unnecessary medical examinations
    - A breach of a standard of practice or code of ethics
  - Determinants
    - Internal: strong personality
    - External:
      - largescale of GP associations
      - No support from the order of physicians
  - Effects
    - Fear
    - Sadness
    - Lack of understanding
  - Coping
    - Talking to relatives
    - Resilience
      - Sharing experiences with colleagues
- Ethical climate
  - Small groups.
  - Informal engagement is important.

# Interview 5

- Moral distress
  - Situations
    - Too much administrative work
    - Different vision in policy in terms of palliative care.
    - Lack of communication
  - Determinants
    - Internal
      - Experience
      - Ambitious
    - External
      - Importance doctor-patient relationship
      - Top-down governmental guidelines
      - Too large GP association
  - Effects
    - Anger
  - Coping
    - Talking to relatives and colleagues
    - Refuse to follow guidelines
- Ethical climate
  - Physician autonomy
  - Weekly open group consultation
  - Time and space for consultation
  - Role of being physician (no other functions).
  - Good palliative policy
  - Colleagues as friends
  - Informal acquaintance.
  - Authenticity

# Interview 6

- Moral distress
  - Triggers
    - Lack of communication
    - Top-down guidelines: powerlessness
    - Intimidation, hierarchy
  - Determinants
    - Internal
      - Perfectionism
      - professional experience
    - External
      - Different roles as GP
      - Other life roles (parent, partner)
      - Group atmosphere
      - Shortage of time and colleagues (other GP’s)
  - Effects
    - Anxiety
    - Loss of control
    - Insomnia
    - Loss of energy
    - spiritual doubt
  - Coping
    - Talking to relatives and colleagues
    - Music
    - Frequent meetings in group association
    - Nature
- Ethical climate
  - Working with group coordinators, psychologists
  - Meetings
  - Good delegation skills
  - Authenticity
  - Regular update vision text
  - Respect

# Interview 7

- Moral distress
  - Triggers
    - Unsubstantiated patient demand
    - Conflict individual vs collective interest
    - Refusal of treatment by patient
    - Insufficient contextual information
    - Incapacity certificates
  - Determinants
    - Physician-related: stress
    - Practice-related: time pressure
    - Financial
    - Inadequate communication
  - Importance
    - Frustration, lack of understanding
    - Impact on functioning
      - Communication
      - Empathy, commitment
      - Medical decisions?
  - Reaction
    - Relativise
    - Adhere to own values
    - Ventilate
    - Leisure
    - Learning from mistakes
- Ethical climate
  - Informal approach
  - Practice atmosphere
  - Broader context vs biomedical view
  - Heightened contextual sensitivity
  - Professional experience
  - Wait and see
  - Relief
  - Patient-centred communication
  - Informing
  - Openness

# Interview 8

- Moral distress
  - Situations
    - Refusal of intervention
    - Demanding patients
    - Unjustified demand for certificates
  - Determinants
    - Solo practitioner
    - Legal uncertainty, legal complexity
    - Contextual constraints (private nursing homes)
    - Work pressure
  - Effects
    - Worse sleep
    - Persistent commitment and even behaviour
    - Considering leaving a job
    - Discouraging career choice
  - Coping
    - Initial: anger
    - Coping mechanisms over time
  - Resignation and acceptance
  - Humour and cynicism
  - Alcohol
  - Creating free time
  - Sleeping sufficiently
  - Ventilating
- Ethical climate
  - Informal
  - Openness
  - Flexible perspective (adjusting opinions)
  - Professional growth (learning from mistakes)
  - Role
    - Ventilating
    - Collegiality, connectedness
    - Understanding

# Interview 9

- Moral distress
  - Triggers
    - (patient stop)
  - Determinants
    - Uncertainty and lack of knowledge
    - Unprecedented situation (covid)
    - Fake news
    - Religious/cultural differences
    - Unclear guidelines
    - Logistical constraints
    - Solo doctor
  - Effects
    - Stress, poor sleep
  - Coping
    - Free time
    - Discussing
    - Informing
    - Professional experience
- Ethical climate
  - Dialogue
  - Alertness, moral sensitivity
  - Awareness of limitations
  - Accessibility
  - Commitment
  - Flexibility
  - Feedback
  - Confidentiality
  - Fellow sufferers contact
    - Emotional release
    - Providing solutions
  - Time ("wait and see")

# Interview 10

- Moral distress
  - Triggers
    - Inappropriate questions from patients
    - Willful incapacity (dementia)
  - Determinants
    - Relationship GPT-GP
      - Hierarchy
      - University doctrine/ zeitgeist
    - Lack of knowledge and experience
    - Lack of openness (adjusting opinions)
    - Consumer behaviour
    - End-of-life care
    - Unclear interests
  - Impact
    - Frustration, irritability, feeling twisted, uncertain
  - Coping
    - Discussing
    - Self-reflection, learning and professional growth
- Ethical climate
  - Awareness of own value framework
  - Expanding perspective
  - Mutually respectful doctor-patient relationship
  - Personal care as a double-edged sword
  - Knowing each other, relationship of trust, safety

# Interview 11

- Moral stress
  - Triggers
    - Danger to third parties
    - Refusal of intervention by patient
    - Patient abandonment
    - Inadequate supply of care
  - Determinants
    - Physician-related
      - Private work stress
      - Conscientiousness and sense of responsibility
      - Personal ethical beliefs
      - Professional experience
      - Feasibility expectations
    - Patient-related
      - Language barrier
      - Unwillingness?
  - Importance
    - Considering job abandonment
    - Guilt, helplessness, relief
  - Coping
    - Active
    - Working part-time
    - Ventilating
      - Formal
      - Informal
    - Updating expectations
- Ethical climate
  - Active approach
    - Structural consultation
  - Vision texts as a guide
  - Protocols and agreements
  - Appointing those responsible
  - Team functioning and atmosphere
  - In-service training, performance interviews
  - Flexibility, modesty, openness
  - Inclusiveness
  - Majority decisions
  - Personal growth and work points
  - Giving time
  - Divided burden and responsibility
  - Counterbalancing self-doubt

# Interview 12

- Moral stress
  - Situations
    - Toxicomaniacs
    - Waiting lists
    - Hierarchy
      - Persons
      - Priorities
- Determinants
  - Poor organization
  - (Time pressure)
  - Financial aspects
  - Informal carers
  - Views of colleagues
  - Care context
- Effects
  - Frustration, lack of understanding, powerlessness
  - Uniform professional functioning
- Coping
  - Ventilating
  - Accepting (good enough)
  - Persistence and sustained commitment
- Ethical climate
  - Active approach
  - Prevention
  - Coach
  - Personality and team dynamics
  - Structural consultation
  - Openness, eagerness to learn
  - Modesty:
    - "I don't know everything, others may have a better view on this"
- Willingness to change
- Protocol for conflict discussions
- Perspective-widening
- Solution-oriented
- Carried responsibility
- Agreements and protocols
- Hierarchy
- Shared vision
- Prior experiences
- Implicit agreements

# Interview 13

- Moral stress
  - Triggers
    - Refusal of intervention by patient
    - Demanding patient
  - Determinants
    - Physician-related
      - Personality
      - Professional experience
        - Coping mechanisms
        - Learning to set limits
      - Lack of time
    - Patient- and context-related
      - Personality
        - "manipulative nature"
        - Psychological problems
      - Toxicomancers
      - Danger to patient
      - Social safety net
      - Unclear demand for help
    - End-of-life situations
    - Sociocultural differences
  - Effects
    - moral residue?
    - Sleep quality
    - "Being outside oneself": frustration, anger
    - Self-doubt
  - Coping
    - Ventilation
    - Dialogue
    - Humour
    - Accepting patient perspective
    - Indicating boundaries
- Ethical climate
  - Informal approach
    - Spontaneity
  - Similar ethical outlook
  - Diversity
  - Trust and familiarity
  - "gut feeling"
  - availability, respect
  - professional competence
  - Equality, no hierarchy
  - Importance
    - Supportiveness and support
    - Different perspectives as added value
- Resilience
  - Recognise moral stress
  - Moral stress as a learning opportunity
    - setting boundaries
  - "Conditional generosity"
