# Supplementary material for: From moral distress to resilient ethical climate among general practitioners: Fostering awareness. A qualitative pilot study
Source: PLoS One. 2024 Aug 30;19(8):e0306026. doi: 10.1371/journal.pone.0306026 (PMC11364290; doi:10.1371/journal.pone.0306026)
Supplement: Attachment — Submitted filename: Thank you for the opportunity to review this interesting paper on moral distress and ethical climate among primary care physicians in Belgium.docx [file pone.0306026.s004.docx]

Thank you for the opportunity to review this interesting paper on moral distress and ethical climate among primary care physicians in Belgium.

I think the topic is interesting, particularly in the way we manage healthcare delivery after the pandemic started. In the present form, the manuscript is not ready for publication. There are some further clarifications needed for some terms used, conceptualization, methods, findings and implications.

Firstly, I feel you need a better justification for interviewing primary care physicians about this topic. I find the results confusing – mainly because there are too many concepts and sub-themes that seem to overlap on another. The results seem to be presented as a list of codes arranged in mind map themes; it gives the impression the data have not been synthesized, lacking in coherent narrative and context. I think it could be presented in a better way. There are some limitations to your study including external validity and you need to write something on reflexivity. Lastly there is an important part in the discussion about what “resilience” means; it can easily be interpreted as “gaslighting”. You can easily blame the person for their moral distress without considering what the organization and health care policies can change to reduce it.

Introduction

What is the difference between “moral distress” and “moral injury”?

Why do you call this a “pilot” study – is this first of a series of studies or a programme of research?

“Resilience” can be viewed negatively in healthcare as a way of blaming the individual for their distress and absolving the organization from responsibility.

The introduction does not explain why primary care physicians are an interesting group to study. Why are they at risk of MD? What assumptions are being made in the last paragraph? Why would increase in group practices to replace single-handed practices warrant investigation into MD?

I would suggest finding literature that support the position that doctors are at risk of moral distress and its consequences eg substance misuse, suicides, mental health issues, job dissatisfaction, attrition from the profession etc. Also give examples of how the “ethical climate” has challenged the workforce eg healthcare scandals, health policies etc.

Study design

The scoping literature review that provided basis for interview guide needs referencing; at least give reasons for the questions you are asking.

Data analysis

Please explain why it is a problem for a person analysing the transcript being able to recognise the interviewee? Especially considering some of them were known to the authors already.

Please explain why you did not ask participants to validate their transcripts.

Results

You mention “in-depth interviews” but you carried out semi-structured interviews.

Micro, meso, macro levels seem to overlap with many sub-themes. Would it not be better to arrange them under these terms?

Micro-level – I think use of “personality traits” might be a bit strong as they suggest an assessment was made using validated instruments. You could use “typologies” instead which gives you some freedom in definition.

Some of the quotes presented here do not seem to illustrate the narrative. For example, being summoned to a police station for something they thought they didn’t do was presented as an example of how some doctors avoided morally distressing situations, or lack of experience causing insecurity; but I am not sure which.

Meso level

How do discussions around finance and guidelines cause resentment?

Macro level

How is the example given about how GPs relating to one another a macro level issue rather than micro or meso? I was also expecting to hear something about healthcare policies here. There were some mention of lack of quality care, lack of facilities and lack of time for administration tasks – but none of these seem to be explained by current health care policies or how health care is organized.

Moral distress – individual consequences.

How is this different from micro level issues?

The first quote given lacked context – why was the participant belittled and laughed at? About what?

Resilient ethical climates – signposts

“Good timing” – I don’t understand why this is a concept for narrative about making time for colleagues; also allow time for deciding what? Things like good practice organization and how tasks are divided and delegated aren’t always related to individual time management but also how the practice is organized.

Peer relationships – isn’t this whole section mainly about peer relationships? Also – this was briefly mentioned in the macro-level point about making time to meet peers?

Again – what is specifically different about “dialogue” to peer relationships? “Respect” – ditto.

Approach to ethical climates – a broad spectrum

The latter paragraph about how some doctors managed dysfunctional ethical climates needs expanding. It also overlaps with a few themes presented later – eg next theme.

Moral resilience short and long term

Short term/individually – so how is this different to the micro level narrative? Also some overlaps with building relationships with colleagues; this point has come up on two other occasions under different themes.

Collectively – see above – why is this not under “relationship with peer” theme?

Long term – professional growth

There is a quote in French – please translate for non-French speakers.

This section appears to have more synthesis of the interview data compared to the others.

I wonder if another way of presenting your data might make better sense. For example, what do your data tell you about why moral distress happen and to whom? Can they be presented as personal factors, interpersonal factors and organizational factors? Similarly, what is the role of ethical climate on moral distress? How do they affect the person, the relationship between people and how do organisations create an ethical climate?

Discussion

I think more explanation is needed about what ethical climate is and how it is related to organizational behavior. For example, a “learning organization” might reflect on adverse events, learn from it and change the way it delivers care or manages people. A toxic organization or one with organizational malaise might reactive differently. The important point is that as an individual, GPs might not be able to influence that. Your suggestions to become “resilient” all focus on how the individual can/should change – but what if the organization is the cause of the problem? The focus on the individual to change might unfairly blame them for not being able to cope. See the British Medical Association (BMA) guidance on moral distress <https://www.bma.org.uk/media/4209/bma-moral-distress-injury-survey-report-june-2021.pdf>

Limitations

The first point seems to be missing – or is it the first sentence? You made a point about “data saturation” – on one hand you felt your data did not reach saturation; on the other, you say another sample would not have changed your findings. I do not believe you reached saturation considering the number of people you interviewed and their backgrounds.

“In depth” interviews mentioned again – you wrote in the methods they were “semi-structured”.

The third point about “shared vision” – this might be because you didn’t ask that question? Or the questions so not naturally lead to this?

External validity – I think this statement is rather brave considering your sample. You rightly mentioned it is contextual so you need to justify why in the context you have chosen, that you are likely to find anything new – are there other published studies that have the same findings? I don’t think your findings are entirely related to the pandemic, in fact there were very few references to it. For example, where is the distress caused by not being able to see a patient in person because of lockdown restrictions? Where is the distress caused by not feeling able to offer any help to some patients who were alone and vulnerable during lockdown?

You also need to add a paragraph about reflexivity. How does your position as GP trainees doing interviews on your peers impact on what they share with you and their responses? How does it affect your analyses?
