# Supplementary material for: From moral distress to resilient ethical climate among general practitioners: Fostering awareness. A qualitative pilot study
Source: PLoS One. 2024 Aug 30;19(8):e0306026. doi: 10.1371/journal.pone.0306026 (PMC11364290; doi:10.1371/journal.pone.0306026)
Supplement: Attachment — Submitted filename: 231213 Response to Reviewers.docx [file pone.0306026.s005.docx]

Response to Reviewers

Dear reviewers

We thank you for taking the time to go through our study thoroughly and providing this valuable feedback.
We addressed all comments below in purple, rewrote our paper and reworked figures in accordance with your remarks.
For better overview, we included tables of concepts in the manuscript as substitutes for mind maps which were provided as figures previously.
We would suggest starting by re-reading the paper in the revised version and subsequently going through our responses upon the questions raised during your review. In our opinion, this will enable checking whether our adaptations to the paper have addressed your suggestions and clarify responses to your questions.

Sincerely

Raf Coremans, Anton Saerens, Yvonne Denier and Jan De Lepeleire

# Reviewer 1

Dear authors,

Thank you very much for the opportunity to read your paper. This article has the potential to

make a valuable contribution to such an important topic. It was well structured and provided

valuable information on a current and essential subject. However, I have some suggestions.

## Introduction

**p.4, lines 47-48**

"The EC then, logically, is a major contextual determinant in the development of a health professional's MD." How? Can we, authors give some examples?

The ethical climate determines what expectations are set for ethical behavior and thus influences ethical decision making. A description of Victor and Cullen was groundbreaking because they try to explain the role of extrapersonal circumstances in ethical decision-making and behavior persons within an organization (Cullen J. The Organizational Bases of Ethical Work Climates. Administrative Science Quarterly. 1988;33:101-25.) More concretely, if a health professional’s conviction of doing the right thing conflicts with the expectations in the organization, this person will experience moral distress. An extreme example would be a ‘progressive’ physician working in a ‘conservative’ institution. Abortion or euthanasia are not accepted whereas the progressive physician might want to explore those care options.
We believe this also works on a material level: certain care facilities that might contribute to good care might be unavailable for a physician.
From personal experience having worked in more and less resilient ethical climates, we can testify that this ethical climate also works more subtly in interpersonal interactions.

In the introduction of the manuscript, we have tried to better clarify this section.

**P.4, lines 57-58**

"Consequently, MD has been linked to individual effects (emotional, physical, spiritual and behavioural), care quality indicators, burnout and job leave intentions (8-16)."

Cited most references seem to be related to nursing contexts. The mechanisms or experiences regarding burnout and job leave intention due to moral distress between physicians and nurses can be different. Please describe or add unique experiences among physicians based on previous studies.

We specified this and made a more clear distinction between health care practitioners and physicians.

- Dzeng E et al. 2020: “Physicians are buffeted by conflicting pressures to reduce costs in some settings while in others, to raise institutional or individual incomes by prescribing or referral practices.
- Dzeng E et al. 2020: when physicians feel compelled to provide potentially harmful or futile treatments, because there is no advance directive or family members disagree, subjecting a patient to perceived futile care near the end-of-life results in moral distress.
- Dzeng E et al. 2020: a patient’s refusal of life-sustaining treatment or desire to seek assistance in dying in states where it is legal may also create moral distress
- Dzeng E et al. 2020: Social injustice: lack of housing and food resulting in markedly worse outcomes in people who are homeless.
- BMA, 2021 Moral distress and moral injury: Recognizing and tackling it for UK doctors
  - Causes
    - Lack of agency to make the best decisions for patients
    - Insufficient resources or non-existent resources to provide care to suitable professional standards
    - Witnessing poor standards of care
    - Practical experience of medical care clashing with ethical standards taught at medical school and doctors’ own personal ethical standards
    - Complicity in wrongdoing
    - End-of-lifecare decisions.
  - Effects
    - Job leave/change
    - Early retirement
    - Working fewer hours
    - …

**p.5, lines 61-64**

As the authors described 'moral resilience' on page 5, lines 61-64, its concept has been more highlighted nowadays. However, readers can be curious about the backgrounds and recent studies on moral resilience among healthcare professionals or in the context of healthcare settings. Furthermore, moral resilience is one of the main concepts of the present research. Thus, please refer to them.

We added the following references:

- Metselaar S, Molewijk B. Fostering moral resilience through moral case deliberation. Nurs Ethics. 2023 Aug;30(5):730-745. doi: 10.1177/09697330231183085. PMID: 37946387.
- Stutzer K, Rodriguez AM. Moral Resilience for Critical Care Nurses. Crit Care Nurs Clin North Am. 2020 Sep;32(3):383-393. doi: 10.1016/j.cnc.2020.05.002. PMID: 32773180.
- Stutzer K, Bylone M. Building Moral Resilience. Crit Care Nurse. 2018;38(1):77-89.
- Denier Y, Gastmans C, Cloet M. Zorgnet-Icuro. Ethisch Advies 21 - Morele Stress in de zorg. Brussel2019.
- Young PD, Rushton CH. A concept analysis of moral resilience. Nurs Outlook. 2017;65(5):579-87

## Methods

Please describe the validity of the present study, like rigour or trustworthiness.

As stated in the previous version, commonly used guidelines for conducting and reporting qualitative research were followed (QUAGOL and SRQR) we followed in this section.

Moreover, the research was based on a scoping literature review, forming the basis of our semi-structured interview guide. For more details, see later. This interview guide was revised after conducting 2 interviews by the 2 principal researchers and discussion of experiences and difficulties encountered during the first interviews with the senior researchers.
We minimized bias through data triangulation: transcripts of interviews were read and concepts distilled individually by the 2 principal researchers individually and then discussed together. A small sample of interviews was read and discussed by all researchers, thus assuring trustworthiness of interview schemes and minimizing researcher bias. In general, conceptual interview schemes did not differ to a large extent between individual researchers. Disagreements between researchers on concept schemes were resolved after discussion. A fitting test of interview schemes and forwards-backwards movement between interviews and concepts schemes was performed. This process was regularly debriefed during intervisions.

## Results

**P21, lines 382-385**

"Leisure time, hobbies, getting enough sleep or using substances are named as strategies to reinforce carrying capacity: We take a drink in the evening to flush out the day. I think that's a normal way of doing things."(D8)"

How does this statement support the description of moral resilience? I wonder what the differences are between the concepts of moral resilience and stress management or coping skills to deal with job stress. They must be distinguished.

From our point of view, stress management is a key adaptive response to moral distress. We describe two aspects of resilience: bouncing back and bouncing forward. Stress management enables to bounce back and not let moral distress or the crescendo effect gain the upper hand. Bouncing forward requires being able to take a distance and being able to see things in a different light. This can only happen after bouncing back.

**P20, lines 369-405**

Especially for those descriptions on page 20, lines 369-405, based on the citation and descriptions of moral resilience in the present manuscript, several readers can beg to differ from the authors' interpretations of moral resilience. I recommend that the authors should reconsider those interpretations of moral resilience or delete these descriptions.

We agree some coping mechanisms with moral distress might not be the most appropriate. However, we interpreted this as a way certain GPs try to bounce back and thus thought it well suited to represent findings from our interviews transparently, without making a judgment about whether this is a beneficial way of bouncing back. As we indicated clearly in the manuscript, this was named as a strategy to reinforce carrying capacity and is not our interpretation of resilient behaviour..
To clarify the concept of moral resilience, we expanded the section in the introduction.

## Discussion

In previous literature, the concepts of professional autonomy and moral courage are the essential factors influencing moral distress. For instance, limited professional autonomy can be one of the organisational constraints at a macro level, and lack of moral courage can be related to how to deal with ethical situations or cope with moral distress. However, it seems that they would not be explored in the present research. I am curious about why and how the authors consider such a gap between the present study and the previous study. Please describe them in the discussion section.

Our intention with this study was to distil the core concepts of moral stress in the very specific context of GPs in Flanders. It was not our intention to look for the concepts and patterns already described previously in other health sector contexts. Yet we also find in this study that restricting professional autonomy can lead to moral distress though coded a bit differently here:

Examples of restrictions of professional autonomy in our study:

- Divergent views of what is perceived as "good care"
  - We think restricted professional autonomy is contained in the concept of “differing visions of good care”
  - During the corona pandemic, the government decided that general practitioners were no longer allowed to visit and treat their patients in residential care centers. One of the GPs testified how it gnawed at him/her not being able to care for his/her vulnerable patients in the residential care. After all, GPs assumed that they were the most appropriate person to handle these patients. Yet they had to watch as other doctors - who did not know their patients at all - took over the care. Sometimes with negative outcomes. (clarified in section moral distress: causes).
- Impending harm to the collective interest.
  - Once again within the theme of Covid-19, some general practitioners experience difficulty with the government's request to test young children for Covid again and again. This is harmful for the children and for de doctor-patient relationship and moreover, it has no added value for the medical policy to be taken afterwards (section about macro-level).
- The meso-level determinant of ethical climate and its sub-determinant hierarchy/equality

In the discussion, we also added a section on moral courage.

# Reviewer 2 (Dr Richard Ma)

Thank you for the opportunity to review this interesting paper on moral distress and ethical climate among primary care physicians in Belgium.

I think the topic is interesting, particularly in the way we manage healthcare delivery after the pandemic started. In the present form, the manuscript is not ready for publication. There are some further clarifications needed for some terms used, conceptualization, methods, findings and implications.

Firstly, I feel you need a better justification for interviewing primary care physicians about this topic.

In the following ways, we have tried to clarify the justification:

- We left away the part about evolution towards collaborative practice as it did not contribute to the general message. This could be interesting to explore in further research, however.
- We described how MD is related to job attrition, burnout, substance abuse in other contexts. We then stated that we have heard of profound effects of MD in GPs.
- We also added a part about studying MD and EC in the specific GP context.
- Being a pilot study, we wanted to explore if and how MD exists in GPs; for this exploratory research, we think the justifications mentioned here, suffices

I find the results confusing – mainly because there are too many concepts and sub-themes that seem to overlap on another.

We agree we used a lot of overlapping concepts; however they are intricately linked; to get a complete understanding we felt we needed to introduce all three concepts of moral distress, ethical climate and moral resilience. Previous research has, for example, concluded that more conceptual work is needed to understand the dynamic interplay between individual moral resilience and the environments where clinicians practice (Rushton & Young 2017 A Concept Analysis of Moral Resilience). So we did try to uncover some relationships.

For clarity and to better aggregate the results, we concluded the results section could be condensed and overlap reduced. We hope this will enhance coherence for the reader.

The results seem to be presented as a list of codes arranged in mind map themes; it gives the impression the data have not been synthesized, lacking in coherent narrative and context. I think it could be presented in a better way.

We rearranged and shortened our results section and replaced mind maps with clearer tables.

There are some limitations to your study including external validity

and you need to write something on reflexivity.
See comments on trustworthiness and rigour previously and remarks later

Lastly there is an important part in the discussion about what “resilience” means; it can easily be interpreted as “gaslighting”. You can easily blame the person for their moral distress without considering what the organization and health care policies can change to reduce it.

Introduction was updated. We had left away a previously written part about how the concept of moral resilience excludes “gaslighting” in the original submission, and reintroduced it now.

## Introduction

What is the difference between “moral distress” and “moral injury”?

We chose to not include another concept as moral injury in the introduction; we think we referred to the concept of moral injury without explicitly naming it.
Moral injury can arise from a moral distress experience. A moral distress event can or cannot lead to permanent damage. If there is permanent mental or physical health damage, or damage to one’s moral integrity, then we can speak of moral injury, a permanent scar y. A moral injury also occurs when an individual reaches a breaking point after accumulation of several morally distressing events (crescendo-effect) described in our introduction. Moral resilience can prevent moral distress from becoming a moral injury; however a moral injury can also lead to moral resilience, albeit with permanent change of how the person’s self-concepts

Why do you call this a “pilot” study – is this first of a series of studies or a programme of research?

Because it is the first study on moral distress in primary care physicians (out of hospital) as far as we know. The goal of this research is to explore themes that have not or barely been explored in primary care. By naming this study a pilot study, we wanted to highlight its explorative character. The goal was to generate some background information, on which a certain awareness, new questions and hypotheses without intent of exhaustiveness could originate.
However, as of today we have no plans to pursue further research in this field.

“Resilience” can be viewed negatively in healthcare as a way of blaming the individual for their distress and absolving the organization from responsibility.

We took this comment into account and updated the section about moral resilience in the introduction.

The introduction does not explain why primary care physicians are an interesting group to study. Why are they at risk of MD? What assumptions are being made in the last paragraph? Why would increase in group practices to replace single-handed practices warrant investigation into MD?

One of the goals of this research was to investigate if and how primary care physicians experience MD. To a specific GP readership, it is interesting to be able to identify and relate to MD situations in the specific GP context than in a more generic study.

We left out the part about increase in group practices as this would divert attention from the main research questions to facilitate reading.

Below we would like to briefly answer why our interest was piqued to investigate MD among GPs:

- General practitioners have long been soloists in Belgium. In recent years they have started working more in partnerships. This implies different (ethical) visions under one roof and can lead to tensions.
- We have witnessed MD in primary care colleagues and personal work experiences.
- Because it is one of the first studies on moral distress in primary care physicians (out of hospital) - as far as we know. Other authors came to the recommendation to study ethical climate in other medical settings (Koskenvuori J, Numminen O, Suhonen R. Ethical climate in nursing environment: A scoping review. Nurs Ethics. 2019;26(2):327-45)

I would suggest finding literature that support the position that doctors are at risk of moral distress and its consequences eg substance misuse, suicides, mental health issues, job dissatisfaction, attrition from the profession etc. Also give examples of how the “ethical climate” has challenged the workforce eg healthcare scandals, health policies etc.

To us, it is a logical hypothesis that if a certain type of health care practitioners (i.e. nurses) are at risk of moral distress, other types of HCP would be too.
We updated our introduction with a few references to doctor’s studies, specifically

- Dzeng E et al. 2020:
  - “Physicians are buffeted by conflicting pressures to reduce costs in some settings while in others, to raise institutional or individual incomes by prescribing or referral practices.
  - when physicians feel compelled to provide potentially harmful or futile treatments, because there is no advance directive or family members disagree, subjecting a patient to perceived futile care near the end-of-life results in moral distress.
  - a patient’s refusal of life-sustaining treatment or desire to seek assistance in dying in states where it is legal may also create moral distress
  - Social injustice: lack of housing and food resulting in markedly worse outcomes in people who are homeless.
- Kherbache et al.
  - External constraints
    - Lack of time
    - Lack of recources
    - Low tolerance for critique
  - Internal constraints
    - Lack of moral sensitivity
    - Lack of capacity for ethical reflection
    - Personality traits and individual coping strategies
  - Clinical situations
    - Prolonging the dying process by having to provide futile care
    - Failure to have an end-of-life conversation
    - Witnessing provision of false hope to patients and their family

As an illustration to how ethical climate have challenged the work force, Vryonides et al conducted a study (Ethical climate and missed nursing care in cancer care units). A clear correlation was found between ethical climate types (as defined by Victor & Cullen) and missed care, where missed care is understood as care that was needed for a patient, but not provided through certain circumstances

## Study design

The scoping literature review that provided basis for interview guide needs referencing; at least give reasons for the questions you are asking.

We added a list of references forming the basis for the interview guide and provided more information about the realization of interviews.

## Data analysis

Please explain why it is a problem for a person analysing the transcript being able to recognise the interviewee? Especially considering some of them were known to the authors already.

This statement refers to third persons reading the transcript not involved in the study. We, as researchers conducted interviews and read transcripts.
As the population from which interviewees were drawn was small, and stories about MD highly personal and potentially recognizable, access to full transcripts by a third person could undermine anonymity which was guaranteed participants and required by the ethical committee approving our study protocol and informed consent protocol. This lack of anonymity could hamper full disclosure and lead to response bias.

Please explain why you did not ask participants to validate their transcripts.

Validation of transcripts is not part of the methodology (QUAGOL) we used.

## Results

You mention “in-depth interviews” but you carried out semi-structured interviews.

We do not see a contradiction in those terms. Using semi-structured interview guides, we assured coherence and conceptual uniformity across individual interviews. By allowing space for additional questions in each interview, not included in the interview guide, we tried to encourage participants to fully disclose, thus enabling a rich picture of individual experiences.

Micro, meso, macro levels seem to overlap with many sub-themes. Would it not be better to arrange them under these terms?

For clarity and to better aggregate the results, we condensed the results section and reduced overlap there. We hope this will facilitate coherence.

**Micro-level**

I think use of “personality traits” might be a bit strong as they suggest an assessment was made using validated instruments. You could use “typologies” instead which gives you some freedom in definition.

We change personality traits to typologies.

Some of the quotes presented here do not seem to illustrate the narrative. For example, being summoned to a police station for something they thought they didn’t do was presented as an example of how some doctors avoided morally distressing situations, or lack of experience causing insecurity; but I am not sure which.

This doctor systematically avoided addressing his or her superior about various situations that went wrong on an ethical level in the institution where he/she worked.

The passage from the manuscript was clarified.

**Meso level**

How do discussions around finance and guidelines cause resentment?

The following examples occurred in our interviews but we did not write them out in full in the manuscript:

- For which medical services can you charge money, and for which services can you not?
- Do we attach more importance to quality for an individual patient or do we mainly want to see as many people as possible in one day. In the first case, you earn little as a doctor, in the second case much more…
- When do we follow guidelines and when do we decide to deviate from them?

**Macro level**

How is the example given about how GPs relating to one another a macro level issue rather than micro or meso? I was also expecting to hear something about healthcare policies here. There were some mention of lack of quality care, lack of facilities and lack of time for administration tasks – but none of these seem to be explained by current health care policies or how health care is organized.

Because government bodies expect doctors to perform many administrative tasks, the essence of the profession – in particular: time for patient contact and time for consultation with colleagues - seems to be suppressed. Of course, one could also say this should be categorized under meso-level, as GPs are free to choose how administration is done; IT tasks can be outsourced.

By merging small municipal general practitioner associations into very large regional associations, doctors are becoming less and less familiar with each other personally. As a result, a large part of interpersonal dialogue disappears. We see the evolution towards individualism and less in-person contact as a sociological evolution of alienation, seen in non-medical contexts as well. As this is a broader sociological phenomenon, we categorized it under macro level.

Often doctors mentioned these phenomena as occurring against the backdrop of very specific government policies. These experiences are the facts that came through from our data/interviews. We also see very similar elements in the Kherbache et al article (see reference list).

**Moral distress – individual consequences.**

How is this different from micro level issues?

This part is about the consequences of moral distress. The part about the micro level is about which determinants on a personal level can lead to moral distress.

The first quote given lacked context – why was the participant belittled and laughed at? About what?

We removed this quote as it did not contribute much as an illustration.

**Resilient ethical climates – signposts**

“Good timing” – I don’t understand why this is a concept for narrative about making time for colleagues; also allow time for deciding what? Things like good practice organization and how tasks are divided and delegated aren’t always related to individual time management but also how the practice is organized.

What we tried to say here: it is important to set aside time for meetings about good practice organizations. From the results we learn this is something for which too little time is taken. This implies that underlying problems are not resolved.
Also, our interviews pointed out that there are good and bad moments to discuss certain things, hence ‘good timing’. However, after further thought, the concept of time/timing was redundant with concepts of practice organization, availability etc so we deleted it

**Peer relationships**

Isn’t this whole section mainly about peer relationships? Also – this was briefly mentioned in the macro-level point about making time to meet peers?

Again – what is specifically different about “dialogue” to peer relationships? “Respect” – ditto.

We integrated all these elements (peer relations, dialogue and respect) into meso-level.

**Approach to ethical climates – a broad spectrum**

The latter paragraph about how some doctors managed dysfunctional ethical climates needs expanding. It also overlaps with a few themes presented later – eg next theme.

In this section we wanted to explore to which degree GPs structurally make time for addressing ethical themes in their practice. However, we rearranged this paragraph under meso-level.

**Moral resilience short and long term**

Short term/individually – so how is this different to the micro level narrative? Also some overlaps with building relationships with colleagues; this point has come up on two other occasions under different themes.

Please find the answer above

Collectively – see above – why is this not under “relationship with peer” theme?

Please find the answer above

**Long term – professional growth**

There is a quote in French – please translate for non-French speakers.

We translated all quotes to English.

This section appears to have more synthesis of the interview data compared to the others.

I wonder if another way of presenting your data might make better sense. For example, what do your data tell you about why moral distress happen and to whom? Can they be presented as personal factors, interpersonal factors and organizational factors? Similarly, what is the role of ethical climate on moral distress? How do they affect the person, the relationship between people and how do organisations create an ethical climate?

As defined in our introduction, an ethical climate includes the whole organizational context that influences decisions and actions with ethical connotations by its employees. Thus, we see ethical climate as a very broad phenomenon with complex generative mechanisms and not merely the presence of ‘people working on an ethical climate’. It exists in every organization, whether people are aware of it or not. It can be influenced by the people of the organization. As such, it is a contextual or meso-level determinant of moral distress. We re-arranged our results to make this more clear.

We don’t explain why MD happens to one person and not the other and how determinants interact in one interviewee. We thought a representation of interviews this way could introduce result-presentation bias and given the small sample population potentially lead to identification of interviewees by readers.

## Discussion

I think more explanation is needed about what ethical climate is and how it is related to organizational behavior. For example, a “learning organization” might reflect on adverse events, learn from it and change the way it delivers care or manages people. A toxic organization or one with organizational malaise might reactive differently. The important point is that as an individual, GPs might not be able to influence that. Your suggestions to become “resilient” all focus on how the individual can/should change – but what if the organization is the cause of the problem? The focus on the individual to change might unfairly blame them for not being able to cope. See the British Medical Association (BMA) guidance on moral distress <https://www.bma.org.uk/media/4209/bma-moral-distress-injury-survey-report-june-2021.pdf>

We defined the ethical climate as the whole organizational context that influences decisions and actions with ethical connotations by its employees, based on an extensive conceptual discussion by Newman et al (2017 Ethical Climates in Organizations: A Review and Research Agenda). In that review, readers can find more elaboration on the phenomenon.
We realize this is a broad and somewhat abstract definition to readers who might not have read background literature; however giving a specific example like you propose would, in our humble view, send the reader into a tunnel vision of ethical climate, which is something we explicitly want to avoid and has been subject to criticism as discussed in Newman et al. We believe one of the major conclusions of our research is that MD in GPs occurs in a web of complexly interlinked micro, meso- (ethical climate) and macro determinants over which one has or does not have power, and can acquire more power over time.

This also counters blaming one for his or her moral distress or lack of resilience.
MD and MR are contextual phenomena over which one only has slight control. It happens to people. We can only enhance understanding of MD and MR, as such enhancing understanding and coping with MD and MR. We don’t think we blame anyone here for MD nor MR, we equip our readership with tools to enhance individual and collective moral resilience, thus reinforcing the resilience of the organisation’s ethical climate. Our bottom line is that resilient ethical climates require resilient care practitioners. An individual can try to change contextual determinants through knowledge and ‘cognitive control’ over those phenomena, thus inspiring his or her actions. We think we described this quite concisely.

**Limitations**

The first point seems to be missing – or is it the first sentence? You made a point about “data saturation” – on one hand you felt your data did not reach saturation; on the other, you say another sample would not have changed your findings. I do not believe you reached saturation considering the number of people you interviewed and their backgrounds.

We believe the main conclusions, concepts and story line would not have changed through more interviews or by multiplying sample size. In fact, the main conclusion to our readership is raising awareness about MD and highlighting the context in which MD arises. A more extensive sample would perhaps have led to a more extensive index of MD causes/determinants but not have changed this message; Specific causes of MD seem to be infinite. Most important is the awareness of a whole context influencing MD that, in our opinion, stimulates readers to wonder what factors are involved, and invites the reader or subsequent research to wonder if different factors or levels than described in this study, are playing in a GP’s specific MD experience.

“In depth” interviews mentioned again – you wrote in the methods they were “semi-structured”.

Please find the answer on this question on page 8.

The third point about “shared vision” – this might be because you didn’t ask that question? Or the questions so not naturally lead to this?

We acknowledge here we might not have reached saturation.
However we did ask that question. Here we refer to our interview guide:

- Does your partnership have a vision or mission of practice to fall back on in such situations? (Organisational culture; shared narrative)
  - Explicit or implicit? Carried vision or "scrap of paper"? (Collective moral motivation, moral character)
  - Is it regularly reviewed?
  - Is it based on values?
  - Are there also people in the organisation who are not believing in this vision?

External validity – I think this statement is rather brave considering your sample. You rightly mentioned it is contextual so you need to justify why in the context you have chosen, that you are likely to find anything new – are there other published studies that have the same findings? I don’t think your findings are entirely related to the pandemic, in fact there were very few references to it. For example, where is the distress caused by not being able to see a patient in person because of lockdown restrictions? Where is the distress caused by not feeling able to offer any help to some patients who were alone and vulnerable during lockdown?

Question about not mentioning detailed causes of MD during the pandemic addressed above – this would not have changed the main lines of our report.
Making a comprehensive list of MD causes was beyond the scope of this pilot study, and probably beyond the scope of any study on the subject. After conducting a certain number of interviews, the main narrative lines did not change much, as mentioned above.
The pandemic came with a certain context of guidelines and administrative burden on GPs, potentially biasing our reporting toward the covid context and less generalizable to a non-pandemic context. We did not state our findings are entirely related to the pandemic.

You also need to add a paragraph about reflexivity. How does your position as GP trainees doing interviews on your peers impact on what they share with you and their responses? How does it affect your analyses?

We briefly tried to mention this in the first paragraph of the limitations part, under “interview bias”.
There are numerous reasons why our position as GP trainees could affect our findings. For example as GP trainees we could have been inclined to base our interview guide, questions and data processing on proper experiences and thus unwillingly exclude or not fully understand MD experiences of elder GPs, biasing our report.
Also more experienced GPs might have withheld certain answers or not shared thoughts in order not to “shock” junior doctors. Etc….
We tried to counter our relative inexperience in this field through regular feedback rounds with our experienced supervisors, JDL and YD.

# General questions

1. Kindly provide a brief and more relevant justification for focusing on primary care physicians in your study.

In the following ways, we have tried to clarify the justification:

- We left away the part about evolution towards collaborative practice as it did not contribute to the general message.
- We described how MD is related to job attrition, burnout, substance abuse in other contexts. We then stated that we have heard of profound effects of MD in GPs.
- We also added a part about studying MD and EC in the specific GP context.

2. Kindly re-evaluate your coding methodology and the resulting themes. Perhaps consider re-arranging your findings in traditional themes and sub-themes, and then correlate these with your mind-map analysis. Perhaps as a form of method triangulation?
We rearranged and condensed findings, as described in more detail previously.
We did data triangulation through the QUAGOL method, where interviews conducted by one researcher were also processed by the other.
We also conducted surveys about moral distress (data not shown) before in-depth interviews as a form of method triangulation. Although those data are not included her, we found that system factors such as administrative burden and responsibility for too many patients quantitatively score highest as determinants of moral distress. Apparent contradictions in causes of moral distress between the survey and interviews teach us that the rich essence of moral stress is only uncovered when in-depth dialogue takes place; questionnaires, in turn, allow a more global overview of the determinants.

3. In your methods section briefly elaborate on issues such as validity, including external validity of the study, attempts to achieve data saturation (or why data saturation could not be achieved). Also briefly describe reliability and trustworthiness of the study data and reflexivity.

We expanded our methods and discussion accordingly. For further detail, please refer to responses above.

4. Then, kindly summarize the strengths and limitations of the study in the sections on " Study limitations".

We updated this section about strengths and limitations. Please refer to our revised manuscript

5. In your Discussion section, kindly elaborate further to distinguish and better characterize 'moral resilience' and 'moral distress' with particular reference to the findings from this study, in the context of other studies in the literature.

By rearranging both our introduction and discussion, we sought to better characterise and define the concepts of MR and MD thus facilitating interpretation of findings.

6. Kindly provide relevant examples of moral distress among physicians/ primary care physicians as opposed to nursing contexts.

We extended our discussion with relation to other care contexts; we think that the determinants of MD in nurses also recur in GPs; their relative importance might however differ; for example, we suppose GPs will encounter less distress through conflicting visions with a hierarchical superior than nurses do. An explicit comparison of both settings could provide more insight in the phenomenon; However a further extension of our discussion in this direction is beyond the scope of this research.

7. Kindly address all other issues , comments, and recommendations by the peer-reviewers.
