# Supplementary material for: From moral distress to resilient ethical climate among general practitioners: Fostering awareness. A qualitative pilot study
Source: PLoS One. 2024 Aug 30;19(8):e0306026. doi: 10.1371/journal.pone.0306026 (PMC11364290; doi:10.1371/journal.pone.0306026)
Supplement: Attachment — Submitted filename: Response to reviewers.docx [file pone.0306026.s006.docx]

Dear reviewers

In response to your new comments, please find our response below.
As non-native speakers we acknowledge having used some words inappropriately.

 "Line 240 - i think you mean "venting" rather than "ventilating". Same in line 475, you use "venting" later in that paragraph.

- We adapted the wording to your suggestion. This was done on line 240, 475 and in Table 5.

Line 270 - table 2 - instead of "job leave" do you mean resignation?

- We adapted the wording to your suggestion. This was done on line 74, 84, and in Table 2.

Line 446 - instead of "qualitative care" do you mean quality of care?

- We adapted the wording to your suggestion. The wording was adapted on line 450, too.

Line 625 - I think "exploratory" might be a better word."

- We adapted the wording to your suggestion.

Apart from those changes, we addressed some remaining punctuation and wording issues.

We thank you for your time and constructive and thorough feedback during this review process and hope this research will contribute valuable insights to GPs and researchers.

Kind regards,

Anton Saerens, Raf Coremans, Yvonne Denier and Jan De Lepeleire
